# Incremental Multi-Step Learning MLP Model for Online Soft Sensor Modeling

**DOI:** 10.3390/s25144303

**Published:** 2025-07-10

**Authors:** Yihan Wang, Jiahao Tao, Liang Zhao

**Affiliations:** 1 College of Artificial Intelligence, Beijing Normal University, Beijing 100875, China; 202211081005@mail.bnu.edu.cn; 2 Key Laboratory of Smart Manufacturing in Energy Chemical Process, Ministry of Education, East China University of Science and Technology, Shanghai 200237, China; y80220186@mail.ecust.edu.cn

**Keywords:** incremental learning, soft sensing model, MVMS-MLP, industrial process

## Abstract

Industrial production often involves complex time-varying operating conditions that result in continuous time-series production data. The traditional soft sensor approach has difficulty adjusting to such dynamic changes, which makes model performance less optimal. Furthermore, online analytical systems have significant operational and maintenance costs and entail a substantial delay in measurement output, limiting their ability to provide real-time control. In order to deal with these challenges, this paper introduces a multivariate multi-step predictive multilayer perceptron regression soft-sensing model, referred to as incremental MVMS-MLP. This model incorporates incremental learning strategies to enhance its adaptability and accuracy in multivariate predictions. As part of the method, a pre-trained MVMS-MLP model is developed, which integrates multivariate multi-step prediction with MLP regression to handle temporal data. Through the use of incremental learning, an incremental MVMS-MLP model is constructed from this pre-trained model. The effectiveness of the proposed method is demonstrated by benchmark problems and real-world industrial case studies.

## 1. Introduction

In process industries like metallurgy, machinery, and chemical production, key variables are often difficult to measure directly, making manual sampling and offline analysis necessary. This method leads to delayed data acquisition and low sampling frequencies, which can hinder process control and optimization efforts. To overcome these limitations, soft sensor modeling has become an essential area of research and application. Through the development of mathematical models to estimate critical variables that are hard to measure directly, soft sensor modeling addresses the drawbacks of traditional methods, thereby enhancing accuracy in process monitoring and control [1,2,3].

In recent years, the integration of deep learning into soft sensor modeling has demonstrated significant potential in feature representation, garnering the attention of numerous researchers. Thuruthel et al. developed synthetic systems that utilize redundant and unstructured sensor topologies embedded in soft actuators, along with vision-based motion capture systems and general machine learning techniques, to develop models [4]. A spatiotemporal attention-based long short-term memory (LSTM) network for soft sensor modeling is proposed by Yuan X et al., representing a substantial advancement in the field. This method effectively identifies key input variables related to product quality at each step while adaptively uncovering hidden states associated with quality across the entire time series, thus improving its capacity to manage complex industrial processes [5]. Similarly, Ma L et al. proposed a multi-step sequence-to-sequence model that leverages the attention LSTM neural network, specifically designed to enhance the performance of soft sensors in industrial processes characterized by strong dynamics and nonlinearity [6]. Chen Y et al. developed the multivariate deep reconstruction neural network, a deep learning model optimized for multivariate time-series prediction, which excels at capturing complex interactions among multiple variables, making it highly effective for process optimization [7]. Ou C et al. introduced a quality-driven regularization method for deep networks, which improves the extraction of quality-related features from industrial process data, aligning these features with process quality goals and enhancing soft sensor performance [8]. Ke W et al. proposed an LSTM-based architecture as a soft sensor approach to address the strong nonlinearity and dynamics inherent in industrial processes, leveraging the capability of LSTM to capture long-term dependencies, thereby enhancing model accuracy and robustness [9]. Yuan X et al. developed an output-related variable-wise weighted stacked autoencoder for hierarchical feature representation, leveraging correlation analysis with the output to identify significant variables. This approach enhances both predictive accuracy and model interpretability [10]. Jia M et al. reviewed the applications of graph neural networks in soft sensing, fault diagnosis, and process monitoring [11].

However, despite these advancements, many models still face challenges in fully capturing the complex temporal dynamics present in industrial time-series datasets, which limit their predictive accuracy and overall performance. Further research is needed to better integrate temporal information to improve the effectiveness of soft sensors in industrial applications. Li Z et al. proposed a quality-driven hybrid neural network for soft sensing in refining processes that leverages wavelet-extracted quality information to guide multi-attribute feature learning, enabling accurate prediction of current quality indicators while preserving historical process characteristics [12]. Jin H et al. developed the MRMRRL method, a deep learning-based soft sensor framework that combines nonlinear relevance-weighted feature extraction, kernel PCA-based redundancy reduction, and layer-wise expansion to improve the relevance of latent features to quality variables while reducing model complexity and enhancing prediction accuracy [13].

In machine learning, incremental learning means the capability of a system to continually acquire and link new knowledge from new data while preserving most of the previously learned information. This capability allows models to update incrementally by learning from small batches of new data without requiring re-training on the entire dataset [14]. This feature is particularly beneficial in scenarios involving large-scale data processing or real-time feedback, where it significantly enhances the adaptability of the model [15,16]. Yan S et al. proposed a two-stage learning method that utilizes dynamically scalable representations, leading to more effective incremental concept modeling [17]. Chi Z et al. introduced a meta-learning-based two-layer optimization framework designed to facilitate incremental learning within the few-shot class-incremental learning setting. This framework extracts incremental task sequences from the base class for training and simulates evaluation protocols to ensure robust learning [18]. Castro F M et al. developed a method that integrates a distillation measure to retain knowledge of previously learned classes, alongside a cross-entropy loss function that aids in learning new classes, thereby balancing the preservation of old knowledge with the acquisition of new information [19]. For image classification, a thorough survey of existing class-incremental learning methods was conducted by Masana M et al., performing extensive experimental evaluations on thirteen different approaches to assess their effectiveness and limitations [20]. Tao X et al. used a neural gas (NG) network to represent knowledge, which preserved and learned the topology of the feature manifold [21]. Liu Y et al. presented adaptive aggregation networks, a novel network architecture that features stable and plastic residual blocks at each level, using ResNet as the base architecture. This design effectively balances the integration of new knowledge with the preservation of previously acquired information, thereby improving the ability of the model to adapt to new data while maintaining the integrity of existing knowledge [22].

In incremental learning, the main challenge lies in incorporating new input data while preserving previously acquired knowledge. To address this challenge, the model must absorb the boundaries introduced by new data and accurately represent new knowledge, ensuring that the original knowledge remains intact. When dealing with complex temporal conditions, industrial processes often span extended periods and involve multiple interdependent sub-processes. These sub-processes are strongly coupled, with upstream activities influencing downstream operations, ultimately impacting key variables. Spatially, the effects of upstream sub-processes propagate through the system, shaping the outcomes of downstream processes. Wang Y et al. propose a multirate autoregressive dynamic latent variable model trained with the EM algorithm and maximum likelihood estimation to effectively capture cross- and autocorrelations in industrial processes, preserving multirate data structure for improved soft sensor prediction accuracy [23].

From a time-series perspective, the data generated comprises a sequence of values recorded continuously and chronologically, including real-time monitoring of variables such as temperature, pressure, and other critical process parameters. This time-series data is crucial for analyzing the dynamic behavior of industrial processes, enabling the identification of trends and potential issues influenced by the interconnected nature of the sub-processes [24,25,26]. At a multivariate level, industrial processes are typically monitored by multiple sensors simultaneously for different features, resulting in multidimensional temporal data. Furthermore, data can be prone to anomalies due to sensor quality issues or environmental factors, causing drift and unstable outliers [27]. Key variables are closely interrelated with other process variables over time and exhibit strong autocorrelation due to the continuous and dynamic nature of operations. These complex and dynamic spatiotemporal characteristics present significant challenges for the soft sensor development of process indicators. Additionally, there is currently no standardized approach for developing soft sensor models using deep learning techniques. Shen B et al. introduce a Gaussian mixture that integrates Gaussian mixture modeling with deep time-series decomposition to handle multimodal process data, extract meaningful temporal patterns, and generate synthetic time series for improved prediction performance in industrial soft-sensing tasks [28].

Interpretable soft sensor models enable engineers and domain experts to understand how input variables influence predicted outputs, facilitating better decision-making, model validation, and fault diagnosis. Cao et al. conducted a comprehensive review of interpretable and stable soft sensor modeling techniques, emphasizing interpretable machine learning and causal discovery methods to enhance model transparency and robustness under dynamic industrial conditions [29]. Jia M et al. propose a physical-anchored graph learning method that leverages graph convolutional and recurrent networks to capture spatial–temporal dependencies in process data, thereby enhancing prediction accuracy and model interpretability based on process knowledge [30].

To address these issues, this study introduces a soft sensor modeling framework called the incremental MVMS-MLP network, specifically designed to improve the adaptability of the soft sensor model by using new samples. The incremental learning framework captures complex temporal dynamics by continuously updating the model with new samples while retaining past knowledge. This allows it to adapt to non-stationary conditions and maintain sensitivity to both short- and long-term dependencies in industrial time series.

The primary contributions of this work are as follows:

(1) A pre-trained MVMS-MLP soft sensor framework is developed to improve the prediction of hard-to-measure variables from industrial time-series data.

(2) An incremental learning mechanism is integrated into the MVMS-MLP model, enabling continuous adaptation to new data without full re-training.

(3) An industrial case study from an MAPD hydrogenation reactor process is conducted to verify the prediction accuracy and adaptability of the proposed method.

This paper is structured as follows: Section 2 discusses the pre-trained soft sensor modeling framework utilizing MVMS-MLP. In Section 3, we delve into the real-time incremental model built upon incremental MVMS-MLP. Section 4 presents an evaluation of the model’s effectiveness and performance, demonstrated through benchmark problems and industrial case studies. Section 5 provides the conclusions and suggests potential directions for future research.

## 2. Proposed Methodology

### 2.1. MLP Model and Algorithm

A multilayer perceptron (MLP) is a forward-propagation neural network model widely used for supervised learning, particularly in labeled multi-step prediction. By utilizing a large number of samples, the MLP learns the nonlinear mapping relationships between inputs and outputs [31,32,33]. It approximates nonlinear functions through the addition of hidden layers, enabling it to capture complex nonlinear dependencies between variables. These hidden layers typically employ nonlinear activation functions, such as Rectified Linear Unit (ReLU), which enhance the model’s adaptabilityto complex decision surfaces.

The MLP is well suited for multi-input and multi-output configurations, enabling it to predict future values for multiple variables simultaneously. The adjustable parameters include the number of hidden layers, the number of neurons per layer, and the choice of optimizer. Achieving an optimal model requires continuous training on the training set to fine-tune these parameters and the overall architecture. The MLP supports end-to-end training, which eliminates the need for feature engineering and allows raw data to be directly processed as input.

To assess the performance of the model, metrics such as mean squared error (MSE), mean absolute error (MAE), and the correlation coefficient with the prediction target are used to measure the alignment of prediction results with the original data. Through the backpropagation of errors after forward propagation, model parameters are optimally improved and refined continuously.

In summary, the MLP exploits its strong nonlinear fitting abilities to perform end-to-end learning of complex relationships between variables, making it one of the most frequently used models for multivariable and multi-step prediction [34,35,36,37].

**Figure 1:**
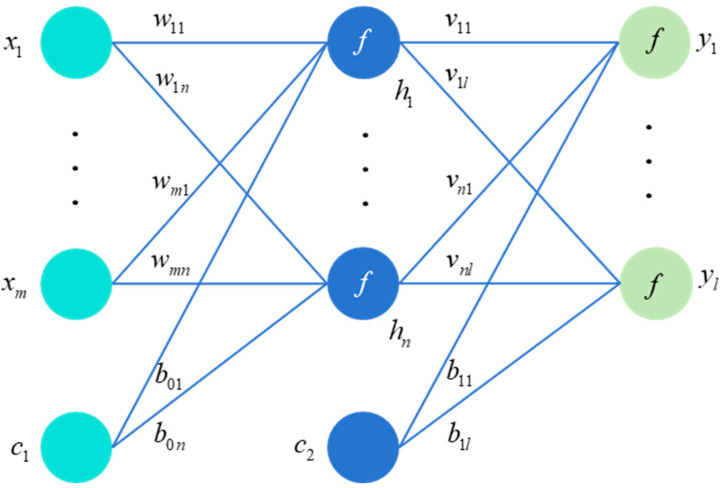
MLP model structure.

In the MLP depicted in Figure 1, the relationship between the input layer *x*, the hidden layer *h*, and the output layer *y* is as follows:

(1) $$h_{1}=f(\sum_{i=1}^{m}w_{1i}x_{i}+c_{1}b_{01})$$

(2) $$h_{n\prime }=f(\sum_{i=1}^{m}w_{n\prime i}x_{i}+c_{1}b_{0n\prime }),\;\;\;n\prime =2,\ldots ,n$$

(3) $$y_{l^{\prime }}=\sum_{j=1}^{n}h_{j}v_{{jl}^{\prime }}+c_{2}b_{{1l}^{\prime }},\;\;\;l^{\prime }=1,2,\ldots ,l$$

Here, *f* is a nonlinear function, also known as an activation function. $n^{\prime }$ and $l^{\prime }$ are indices of the number of neurons in hidden and output layers. $c_{1}$ and $c_{2}$ are constants which are set to 1 in this work.

The aim is to minimize the prediction error of the MLP for samples. For any sample, the error can be defined as $Loss={\left(y-\hat{y}\right)}^{2}$. If there are *N* samples in the training set, then it is necessary to sum up the errors of each sample and minimize them. In Equations (1)–(3), the variables to be optimized are *w*, *v*, and *b*. And it is an unconstrained problem, so it can be solved through gradient algorithms.

Write the expression in matrix form and take the derivative to obtain $\frac{\partial h}{\partial w^{T}}=f^{\prime }\times x$, $\frac{\partial \hat{y}}{\partial h}=v^{T}$, and $\frac{\partial Loss}{\partial \hat{y}}=y-\hat{y}$. According to the chain rule of differentiation,

(4) $$\frac{\partial Loss}{\partial w^{T}}=\frac{\partial Loss}{\partial \hat{y}}\times \frac{\partial \hat{y}}{\partial h}\times \frac{\partial h}{\partial w^{T}}=\left(y-\hat{y}\right)\cdot v^{T}\cdot f^{\prime }\cdot x$$

This is error backpropagation, which is the process of gradient calculation, gradually derived from the back to the front.

### 2.2. MVMS-MLP

In multivariate multi-step prediction, a predictive regressor is combined with multiple input variables and output variables to enable multi-step forecasting. The input and output variables are usually modeled with a predictive regressor, which is then used for multi-step prediction [38,39]. The term ‘multivariate’ indicates that the model predicts the values of multiple variables simultaneously. ‘Multi-step’ refers to the prediction of the future trends of these variables over a sequence of time steps, which is known as multi-step prediction. This method involves forecasting across multiple time steps, allowing for a comprehensive understanding of the trend of variables. In this work, the symbol ${\left(y_{t}\right)}_{t=1}^{T}=\left(y_{1},y_{2},\ldots ,y_{T}\right)$ is utilized to indicate non-predictive and target series in the past T time slots. $x_{t}=\left(x_{t}^{1},x_{t}^{2},\ldots ,x_{t}^{n_{in}}\right)$ is a vector with $n_{in}$ non-predictive variables at time step *t*. Meanwhile, the symbol $y_{t}$ is employed to represent the corresponding target variable at time *t*. The output of the prediction model is an estimation of the target variable of the subsequent $n_{out}$ time steps after *T*, denoted as ${\left({\hat{y}}_{t}\right)}_{t=T+1}^{t=T+n_{out}}=\left({\hat{y}}_{T+1},{\hat{y}}_{T+2},\ldots ,{\hat{y}}_{T+n_{out}}\right)$. The symbol $n_{out}$ ($n_{out}$ > 1) is generally called the forecast horizon, which is a variable based on task requirements. Hence, the problem is formulated as nonlinear mapping from input series ${\left(x_{t}\right)}_{t=1}^{T}$ and target series ${\left(y_{t}\right)}_{t=1}^{T}$ in the history to the estimation of the future value ${\left({\hat{y}}_{t}\right)}_{t=T+1}^{t=T+n_{out}}$, $n_{out}$ time steps ahead:

(5) $${\left({\hat{y}}_{t}\right)}_{t=T+1}^{t=T+n_{out}}=F\left({\left(x_{t}\right)}_{t=1}^{T},{\left(y_{t}\right)}_{t=1}^{T}\right)$$

where *F* is the nonlinear mapping function. Replacing the F function with the MLP model yields

(6) $${\left({\hat{y}}_{t}\right)}_{t=T+1}^{t=T+n_{out}}=MLP\left({\left(x_{t}\right)}_{t=1}^{T},{\left(y_{t}\right)}_{t=1}^{T}\right)$$

**Figure 2:**
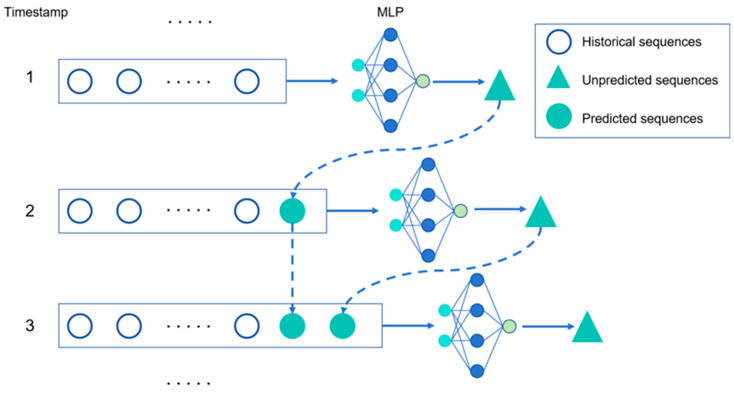
Training set update process of incremental learning in MVMS-MLP.

The structure of the MVMS-MLP network model is shown in Figure 2, where the timestamps 1 to 3 are the steps of changes of training and predicting data. Of course, not only MLP models but also other machine learning models such as LSTM and GRU can be used.

## 3. Incremental MVMS-MLP

### 3.1. Incremental Learning

Incremental learning of data requires the use of incremental online gradient descent algorithms, with the basic idea of the following calculations:

(1) Calculate errors: e=y−f(x;w), where *y* is the label of the new data sample, and f(x;w) is the predicted value of the network model for new data.

(2) Calculate gradient: $\frac{\partial e}{\partial w}$.

(3) Update parameters using learning rate η: $w=w-\eta \frac{\partial e}{\partial w}$.

The loss function is mean square error and is given as follows:

(7) $$L(w)=\frac{1}{2n}{\sum \left(y-f(x;w)\right)}^{2}$$

Use i to represent the index of new data samples; for each new data sample $\left(x_{i},y_{i}\right)$, the loss function is rewritten as

(8) $$L_{i}(w)=\frac{1}{2n}{\sum \left(y_{i}-f(x_{i};w)\right)}^{2}$$

According to the gradient descent algorithm, the direction of the weight update is the gradient of the loss function relative to w:

(9) $$\nabla L_{i}(w)=-(y_{i}-f(x_{i};w))x_{i}$$

According to online algorithms, several new data samples update their weights once:

(10) $$w=w-\eta (y_{i}-f(x_{i};w))x_{i}$$

Repeating this process can achieve incremental online gradient descent.

### 3.2. Procedure of Incremental MVMS-MLP

**Figure 3:**
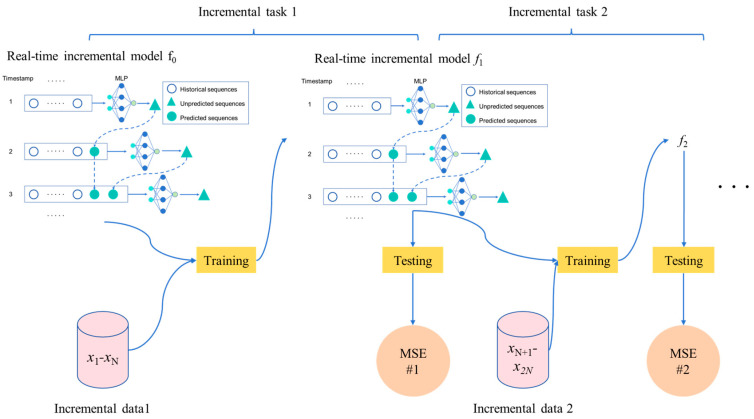
Structure of incremental MVMS-MLP.

The structure of the incremental MVMS-MLP model is shown in Figure 3. $X_{n}\in R^{m\times \ln}$ are the feature vectors of new incremental samples, and $Y_{n}\in R^{\ln}$ ln are the corresponding new incremental samples. *l_n_* is the number of new incremental samples. For example, the sample of the first increment in Figure 3 is $X_{1}-X_{N}$, the sample of the second increment is $X_{N+1}-X_{2N}$, and so on. The pre-trained MVMS-MLP model is called the real-time incremental model $f_{0}$. Add new incremental samples $X_{1}-X_{N}$ to model f_0_ and train to obtain the real-time incremental model $f_{1}$. Then, use test set samples to test the f1 model and get the indicator MSE #1, and continue incremental learning according to the above process. Each incremental learning session is compared to a task, such as incremental task 1, incremental task 2, etc. In this way, the incremental MVMS-MLP model proposed in this work can be obtained, as shown in Figure 3.

The loss function $Loss_{1}$ of incremental task 1 is set as

(11) $$Loss_{1}=L\left(f_{0}(X_{1}-X_{N}),(Y_{1}-Y_{N})\right)$$

(12) $$f_{0}(X_{1}-X_{N})=MVMS-MLP_{0}(X_{1}-X_{N})={\left({{\hat{y}}_{t}}^{1-N}\right)}_{t=T+1}^{t=T+n_{out}}$$

where $f_{0}(X_{1}-X_{N})$ represents the predicted value of the *MVMS-MLP*_0_ model. $Y_{1}-Y_{N}$ represents the corresponding measured values.

So, likewise, the loss function $Loss_{2}$ is easily derived:

(13) $$Loss_{2}=L\left(f_{1}(X_{N+1}-X_{2N}),(Y_{N+1}-Y_{2N})\right)$$

(14) $$f_{1}(X_{N+1}-X_{2N})=MVMS-MLP_{1}(X_{N+1}-X_{2N})={\left({{\hat{y}}_{t}}^{N+1-2N}\right)}_{t=T+1}^{t=T+n_{out}}$$

where $f_{1}(X_{N+1}-X_{2N})$ represents the predicted value of the *MVMS-MLP*_1_ model. $\left(Y_{N+1}-Y_{2N}\right)$ represents the corresponding measured values.

In the incremental learning framework, the useful information contained in old samples is kept in the model, which is used as a pre-trained model for incremental learning when new samples are collected.

## 4. Applications

This section details the application of the incremental MVMS-MLP model on benchmark and industrial time-series datasets, to thoroughly evaluate the predictive and generation capabilities of the proposed method. The performance of the incremental MVMS-MLP model was rigorously evaluated using two widely recognized metrics: mean squared error (MSE) and mean absolute error (MAE). These metrics were given to provide a comprehensive assessment of the model’s accuracy and reliability in capturing the underlying patterns in the data and its ability to generalize across different scenarios.

(15) $$MAE=\frac{1}{n}\sum_{i=1}^{n}\left|y_{i}-{\hat{y}}_{i}\right|$$

(16) $$MSE=\frac{1}{n}\sum_{i=1}^{n}{\left(y_{i}-{\hat{y}}_{i}\right)}^{2}$$

Here, n is the number of samples; $y_{i}$ is the measured value; and ${\hat{y}}_{i}$ is the predicted value.

### 4.1. Benchmark Dataset

To assess the effectiveness of the incremental MVMS-MLP algorithm suggested in this study for industrial applications, a widely recognized industrial time-series dataset—the Sulfur Recovery Unit (SRU) dataset from Chiyoda Chemical [40]—is utilized firstly. This dataset contains extensive operational data from the SRU, capturing various key parameters involved in the sulfur recovery process. For predicting the H_2_S concentration in the tail gas of Line 4 in the SRU, historical data was retrieved from the factory’s database, and five relevant variables were selected for analysis. The dataset provides detailed time-series data with a 1 min sampling interval. The feature and target variables from the SRU dataset are outlined in Table 1.

The method used in this study for partitioning the industrial time-series dataset is based on the chronological order of data collection. The time-series data is divided into three segments: the initial 40% is designated as the pre-training set, the next 40% is utilized for incremental learning (incremental set), and the final 20% is used as the test set for final evaluation. This approach ensures that the temporal sequence of the data is preserved, aligning with the requirements of industrial processes.

The experiment was conducted on a computer equipped with an Intel Core i9-12900H processor, 16 GB of memory, and an NVIDIA GeForce RTX 3060 Laptop GPU. The incremental MVMS-MLP model uses a three-layer architecture: the input layer consists of 6 neurons, the hidden layer has 12 neurons, and the output layer contains 1 neuron. The learning rate is set to 0.001, and the Adam optimizer is employed for training.

The benchmark validation results of the incremental MVMS-MLP model are presented in Table 2.

Through the use of the pre-trained MVMS-MLP model on the first 4000 min of data, the MSE for predicting H2S in the testing set was slightly above 0.0008, as reported in the original paper [39], and was compared with that for the MVMS-LSTM and MVMS-GRU models. However, after initiating incremental learning on the pre-trained model, the MAE and MSE for H_2_S in the testing set decreased significantly. For instance, when the real-time incremental sample size is 500 min, resulting in six incremental learning sessions and a total incremental sample size of 3000 min, and the learning rate is set to 0.0015, the MSE for H_2_S in the test set drops to 0.000469, nearly 50% lower than in the original model. Furthermore, when the real-time incremental sample size is 1500 min, with two sessions of incremental learning, and the learning rate is set to 0.005, the MAE for H2S in the test set decreases to 0.11, and MSE decreases to 0.000257, almost 70% lower than in the original model, demonstrating a significant improvement. These results indicate that the incremental neural network model proposed in this paper performs exceptionally well in predicting industrial time-series datasets.

**Figure 4:**
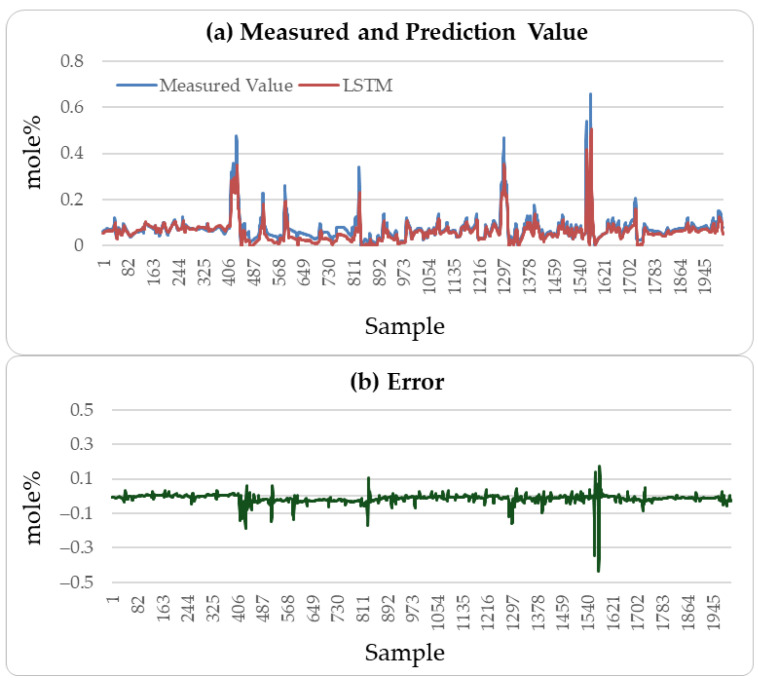
H_2_S prediction results of testing set: LSTM method.

**Figure 5:**
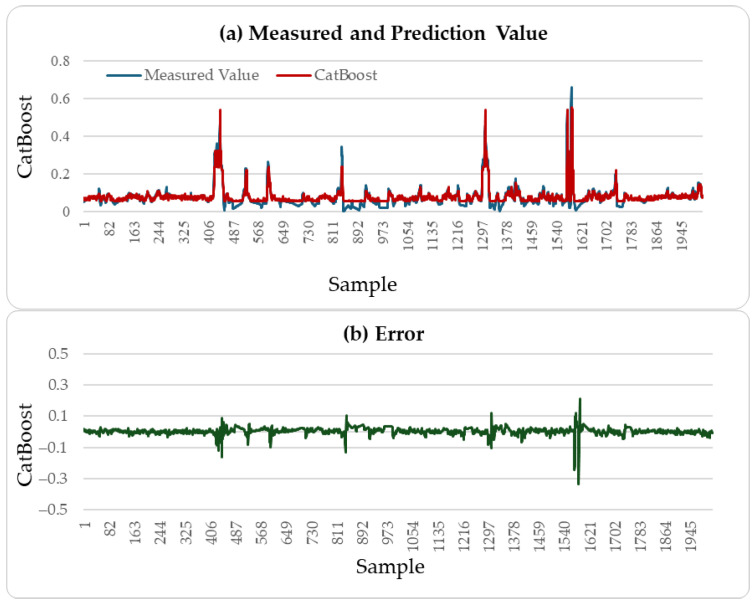
H_2_S prediction results of testing set: CatBoost method.

**Figure 6:**
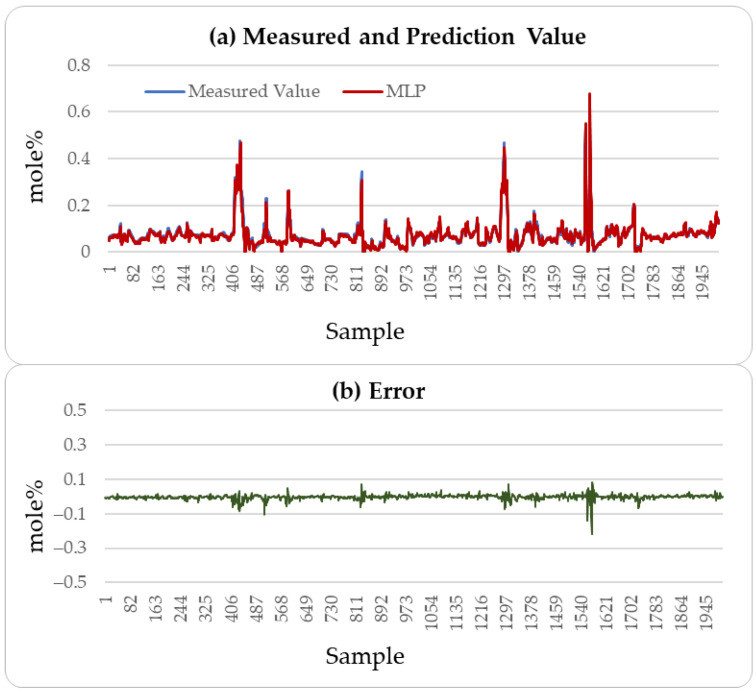
H_2_S prediction results of testing set: incremental sample size of 1500 and incremental learning rate of 0.005.

Figure 4, Figure 5 and Figure 6 illustrate the comparison between the predicted H_2_S values and the actual values over the subsequent 2000 min of the LSTM, CatBoost, and the proposed incremental MVMS-MLP methods. It can be seen that the proposed method has better predictive ability than the LSTM and CatBoost methods. However, when using LSTM and CatBoost methods as the basic model of incremental learning, it can also achieve better performance than single-step learning.

### 4.2. MAPD Soft Sensor Development

#### 4.2.1. MAPD Hydrogenation Reactor Process

In industrial chemical production, propylene is a crucial raw material, extensively used in the manufacture of organic chemical products such as polypropylene, acetone, and isopropanol. The primary component of the C3 fraction produced by the cracking furnace is propylene; however, it also contains impurities such as methylacetylene (MA) and propadiene (PD) [41,42]. These impurities significantly impact the subsequent processes and the quality of the final products, making their real-time prediction and removal essential.

Liquid-phase catalytic hydrogenation technology has become the preferred process for removing impurities in MAPD hydrogenation due to its simplicity, straightforward process flow, mild reaction conditions, and excellent stability. The process flow of the MAPD hydrogenation unit is as follows: C3 fractions are heated by the feed preheater and mixed with circulating hydrogen before entering the hydrogenation reactor for catalytic hydrogenation. After reaction, the mixture is cooled with a circulating cooler and sent to a gas–liquid separation tank. Following the separation of the liquid phase, propylene is forwarded to the subsequent distillation unit, whereas hydrogen-rich tail gas is recycled.

To prevent the reactor from losing temperature control due to excessive heat release during the hydrogenation reaction, a portion of the circulating stream is extracted from the circulation pump outlet, pre-cooled, and diluted with fresh feed through a preheater. Hydrogen and circulating stream are mixed with the feedstock in a flow-proportional control manner, ensuring that the concentration of MAPD at the reactor outlet does not exceed 500 ppm.

**Figure 7:**
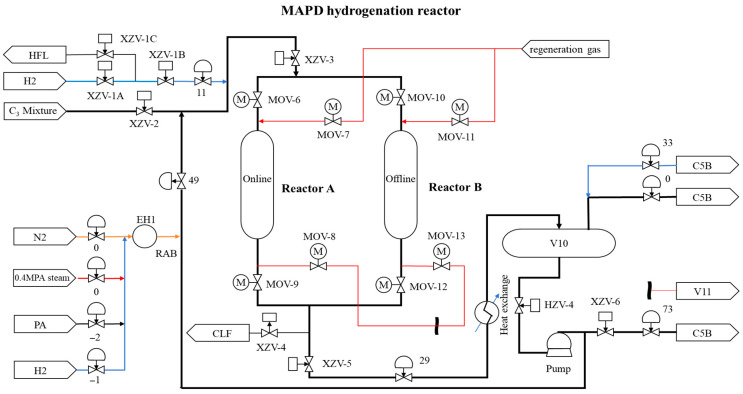
Flowchart of MAPD hydrogenation reactor.

The sampling intervals for the process are set at 5 min, with the average value calculated every five minutes. The MAPD hydrogenation reactor is depicted in Figure 7 with detailed descriptions, unit specifications, and other relevant information provided in Table 3.

#### 4.2.2. Auxiliary Variable Selection

The selection of auxiliary variables is essential to determining the structure and output of a soft sensor model since it determines the matrix of input information. Prior knowledge is generally used to identify auxiliary variables at the beginning of this process. Afterward, statistical methods and analytical techniques are applied to screen and refine these variables, ultimately choosing the most effective set.

The number of auxiliary variables should be determined considering several factors, including the number of variables to be estimated, the degree of process freedom, measurement noise, and the level of model uncertainty. These factors ensure that the model maintains a high level of predictive capability and adaptability. Furthermore, detection points are typically placed based on the dynamic characteristics of the process, with data collection focused on key dynamic features.

By following these guidelines, it is possible to effectively select appropriate auxiliary variables, enabling the construction of a high-performance and reliable soft-sensing model.

Variable normalization is an essential data pre-processing method that adjusts the range of different variables to a common scale, ensuring consistent comparisons and proper handling during data analysis and model training. In soft sensor modeling, the diversity of sensing device types often results in significant dimensional differences among various auxiliary variables. These disparities can obscure variation patterns, weaken the explanatory relationships between auxiliary and dominant variables, and even destabilize gradient calculations during neural network training.

To mitigate these issues, effective data normalization methods must be implemented during pre-processing. This involves mapping the measured values of auxiliary variables to a consistent dimensional scale, thereby eliminating the effects of magnitude differences. The Pearson correlation coefficient helps select auxiliary variables in soft sensor modeling by measuring their linear relationship with the dominant variable. The selected Pearson coefficients in the MAPD soft sensor modeling are shown in Table 4.

#### 4.2.3. Model Architecture

This method of partitioning the soft sensor dataset aligns with the approach used during the algorithm testing process. This consistency ensures that the model fully accounts for the temporal characteristics and trends of the data during training and testing across different stages. The incremental neural network model used for soft sensor development also mirrors the model employed in model testing, utilizing the same network structure. Specifically, the input variables consist of fresh C3 and the inlet MAPD concentration, while the output is the outlet MAPD concentration. The input sequence length is set to 12, meaning that each input sequence includes data from the previous 12 sample points to capture historical information. As the output sequence length is set to 6, the model predicts the next six sample points. By maintaining consistency in dataset partitioning and network models, the soft sensor model can ensure uniform standards throughout various stages of training and testing, thereby facilitating a more accurate evaluation of the approximation and generalization abilities of the model.

#### 4.2.4. Result Analysis

The soft sensor results for the outlet MAPD concentration are presented in Table 5. Using the first 6000 data points, corresponding to the pre-trained MVMS-MLP model trained on the initial 30,000 min of data, the MSE for the MAPD concentration of the testing set was 6647.8. This was compared with the results from the MVMS-LSTM and MVMS-GRU models. During incremental learning on the pre-trained model, the MAE and MSE for MAPD concentrations began to decrease significantly.

For example, with 500 real-time incremental samples for each incremental learning session—with learning conducted over 12 sessions, resulting in a total of 6000 incremental samples—the MSE for the MAPD concentration of the testing set dropped to 3424 when the learning rate was set to 0.01, effectively halving the MSE of the testing set of the pre-trained model. Similarly, using 1500 participants in one incremental learning session, with four such sessions, the MAE of MAPD concentration in the testing set was 46.7, and that of MSE was 3844, showing significant improvement in MAPD concentration prediction.

**Figure 8:**
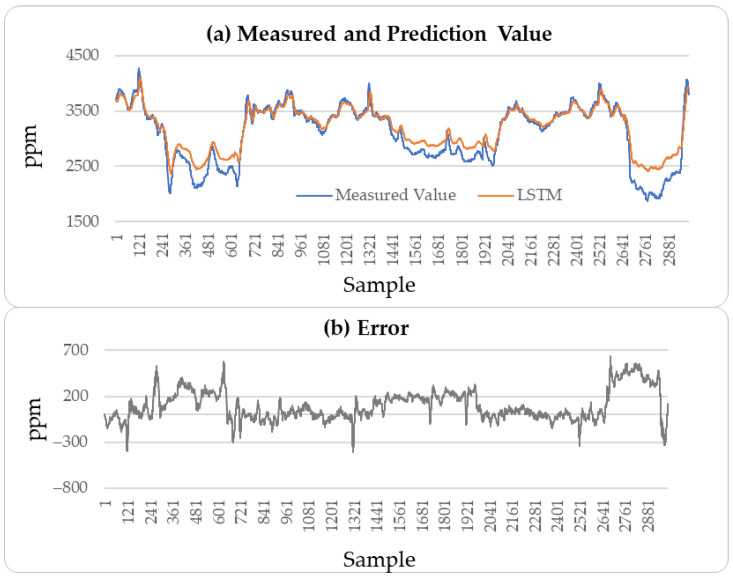
MAPD prediction results of testing set: LSTM method.

**Figure 9:**
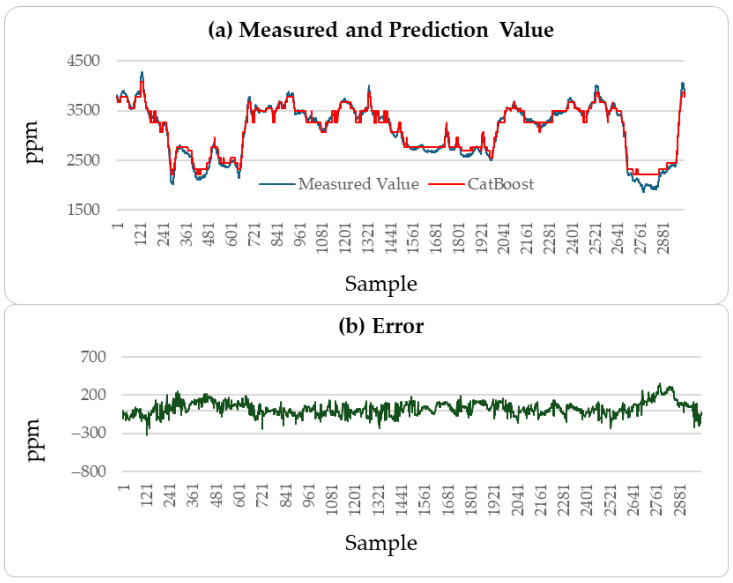
MAPD prediction results of testing set: CatBoost method.

**Figure 10:**
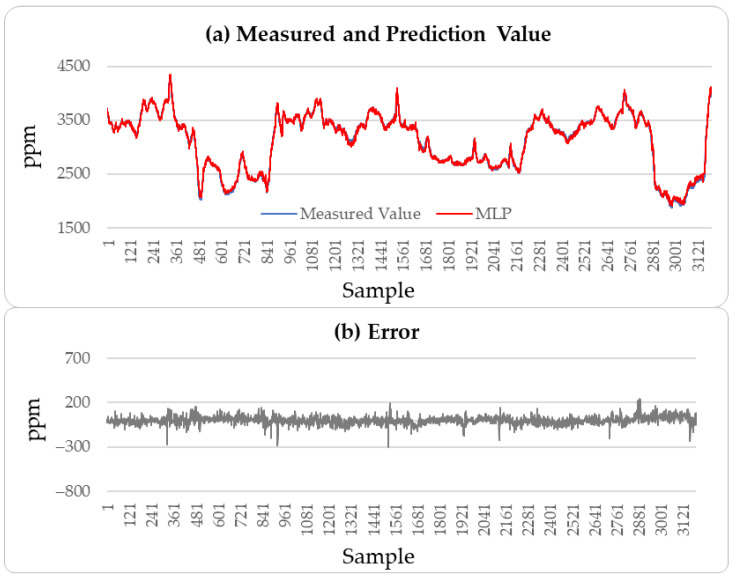
MAPD prediction results of testing set: incremental sample size of 500 and learning rate of 0.01.

These results indicate that the incremental neural network model proposed in this paper performs well in predicting soft sensor data collected from industrial processes. A comparison of the predictive performance of the LSTM, CatBoost, and incremental neural network on the outlet MAPD concentration of the testing set with the measured values was conducted, resulting in a prediction of 3000 outlet MAPD concentration data points, as shown in Figure 8, Figure 9 and Figure 10.

From Figure 8, Figure 9 and Figure 10, it can be concluded that the proposed method has better generation ability to predict the outlet MAPD concentration of the reactor than the LSTM and CatBoost methods. This is because the incremental learning framework utilizes the newest information of the samples to improve the performance of the soft sensor models.

## 5. Conclusions

This paper presents a new framework, incremental MVMS-MLP, specifically designed for soft sensor modeling under complex temporal conditions. Initially, the framework combines multivariable multi-step prediction with a multilayer perceptron to develop the MVMS-MLP model. Next, an incremental learning strategy is integrated into the MVMS-MLP model, resulting in incremental MVMS-MLP, which is effective in processing industrial time-series data through incremental learning. Finally, experimental results from a benchmark problem and an industrial case study demonstrate that the incremental MVMS-MLP model delivers more accurate and adaptive performance.

Although incremental learning is applied in this study in a relatively simple manner, limited to samples, there is potential for future research into more advanced incremental learning strategies. Additionally, although this study employs the MLP model, other models such as LSTM, CatBoost, and the newest machine learning methods could be considered for further research. Furthermore, the proposed soft sensor modeling method ignores outliers or noise data, limiting its application potential. The proposed incremental learning framework will be integrated with data pre-processing methods for industrial soft sensor systems.

## Figures and Tables

**Table 1:** The feature variables and target variables of the SRU.

Auxiliary Variables					Dominant Variable
MEA GAS (Nm^3^/h)	AIR MEA1 (Nm^3^/h)	AIR MEA 2 (Nm^3^/h)	AIR SWS (Nm^3^/h)	SWS GAS (Nm^3^/h)	H_2_S (mol%)

**Table 2:** Benchmark validation results of the incremental MVMS-MLP.

	Single Incremental Sample	Incremental Learning Times	Total Incremental Samples	Incremental Learning Rate	H_2_S MAE	H_2_S MSE
Original paper model						0.0008
LSTM			4000		0.015	0.00065
CatBoost			4000		0.0144	0.0006
GRU			4000		0.0154	0.00079
MVMS-MLP			4000		0.02	0.001
Incremental MVMS-MLP	250	12	3000	0.001	0.0187	0.00085
	250	12	3000	0.0015	0.0183	0.000849
	250	12	3000	0.002	0.0179	0.00084
	500	6	3000	0.001	0.014	0.00049
	500	6	3000	0.0015	0.0126	0.000469
	500	6	3000	0.002	0.011	0.00045
	1000	3	3000	0.001	0.02	0.00075
	1000	3	3000	0.0015	0.017	0.00059
	1000	3	3000	0.002	0.016	0.0005
	1500	2	3000	0.001	0.0117	0.000277
	1500	2	3000	0.002	0.012	0.000266
	1500	2	3000	0.005	0.011	0.000257

**Table 3:** Description of variables for MAPD hydrogenation reactor.

Tag Description	Unit
Fresh C_3_	t/h
Cycle C_3_	t/h
Hydrogen flowrate	kg/h
Outlet temperature	°C
Outlet pressure	MPa
Cycle C_3_ temperature	°C
Outlet online analysis of hydrogen concentration	ppm
Outlet online analysis of propylene	%
Outlet online analysis of MA	ppm
Outlet online analysis of PD	ppm
Inlet online analysis of propylene	%
Inlet online analysis of MA	%
Inlet online analysis of PD	%

**Table 4:** Pearson correlation coefficients of auxiliary and dominant variables.

	Auxiliary Variables		Dominant Variable
Pearson coefficient	Fresh C3 (t/h)	Hydrogen (kg/h)	Outlet MAPD (ppm)
Fresh C3 (t/h)	1.000000	0.692936	0.389205
Hydrogen (kg/h)	0.692936	1.000000	0.215326
Outlet MAPD (ppm)	0.389205	0.215326	1.000000

**Table 5:** The exported MAPD soft measurement results of incremental MVMS-MLP.

	Single Incremental Samples	Incremental Learning Sessions	Total Incremental Samples	Incremental Learning Rate	Outlet MAPD MAE	Outlet MAPD MSE
MVMS-LSTM			6000		54.17	6587
MVMS-CatBoost			6000		71.75	8395
MVMS-GRU			6000		59.5	6047
MVMS-MLP			6000		52	6448
Incremental MVMS-MLP	500	12	6000	0.002	50.75	4961
	500	12	6000	0.005	45.2	3511
	500	12	6000	0.01	44.7	3434
	1000	6	6000	0.001	49.9	4606
	1000	6	6000	0.002	51.9	4589
	1000	6	6000	0.003	47.25	4485
	1500	4	6000	0.002	55.7	5589
	1500	4	6000	0.005	51.8	4890
	1500	4	6000	0.01	46.7	3844

## Data Availability

The original contributions presented in this study are included in the article. Further inquiries can be directed to the corresponding author.

## Abbreviations

MVMS-MLP	Multivariate Multi-Step Multilayer Perceptron
MAE	Mean Absolute Error
MSE	Mean Squared Error
SRU	Sulfur Recovery Unit
MAPD	Methylacetylene–Propadiene
LSTM	Long Short-Term Memory
GRU	Gated Recurrent Unit
ReLU	Rectified Linear Unit
Adam	A Stochastic Gradient Descent Optimization Algorithm
H2S	Hydrogen Sulfide
C3	Propylene Fraction
